# Factors associated with mammogram and Papanicolaou testing among Mexican American older women

**DOI:** 10.1177/17455057261421727

**Published:** 2026-02-19

**Authors:** Emma Rowlinson, Soham Al Snih

**Affiliations:** 1 John Sealy School of Medicine, The University of Texas Medical Branch, Galveston, USA; 2 Department of Population Health and Health Disparities, School of Public and Population Health, The University of Texas Medical Branch, Galveston, USA; 3 Division of Geriatrics Medicine, Department of Internal Medicine, The University of Texas Medical Branch, Galveston, USA; 4 Sealy Center on Aging, The University of Texas Medical Branch, Galveston, USA

**Keywords:** Mexican American, Hispanic, older adults, women, cancer screening, mammography, Papanicolaou test

## Abstract

**Background:**

Mammogram and Papanicolaou (Pap) smear tests are essential screening to detect breast and cancer cervical, respectively.

**Objective:**

Identify predisposing, enabling, and need factors associated with mammography and Paptest screenings among older Mexican American women over time.

**Design:**

Longitudinal study of 912 Mexican American women aged ⩾ 67 years.

**Methods:**

Participants were assessed five times (1995/1996–2007/2008). Independent variables were based on the Andersen’s Behavioral Model of Health Services, including predisposing factors (e.g., age of menopause), enabling factors (e.g., financial strain), and need factors (e.g., medical conditions). Outcomes included having a mammogram in the past 2 years and a Pap test in the past three. Generalized Estimation Equation models estimated the odds ratio (OR) and 95% confidence interval (CI) for receiving a mammogram, Pap test, or both based on these factors.

**Results:**

Higher education (OR = 1.04, 95% CI = 1.01–1.07), physician visits (OR = 1.85, 95% CI = 1.33–2.56), hypertension (OR = 1.26, 95% CI = 1.04–1.51), arthritis (OR = 1.31, 95% CI = 1.07–1.60), and greater handgrip strength (OR = 1.02, 95% CI = 1.01–1.04) were associated with greater odds of receiving both a mammogram and Pap. Older age and early menopause (OR = 0.96, 95% CI = 0.94–0.98 and OR = 0.71, 95% CI = 0.58–0.89, respectively) were associated with lower odds of receiving both tests. Spanish interview (OR = 0.71, 95% CI = 0.56–0.91) and financial strain (OR = 0.83, 95% CI = 0.70–0.99) were associated with lower odds of receiving a Pap test and mammogram test, respectively over time.

**Conclusion:**

Language barriers and financial constraints reduce cancer screening rates among Mexican American women. Cultural tailored care and improved access, such as bilingual clinics and mobile screening are needed to address these gaps.

## Introduction

Breast cancer is a significant health concern amongst women in the United States, affecting about 13% over their lifetime; however, efforts to increase awareness and early detection are made through mammography screening.^1^ The incidence of breast cancer varies by age and the highest incidence has been reported to be at 475.8 per 100,000 among women of 70–74 years.^1^ To maximize screening benefits, medical groups have created guidelines focusing on this age group. The US Preventative Task Force (USPSTF) recommended mammography for women ages 40–74 biennially, an update from the previous ages of 50–74.^2,3^ The American College of Obstetrics and Gynecology (ACOG) and the American Cancer Society (ACS) recommend mammography every 1–2 years from ages 40 to 74.^4,5^ ACOG and ACS also recommend practicing shared decision-making with patients older than 75 to potentially continue screening until <10 years of their life expectancy.^4,5^

Mammograms are a cost-effective method shown to reduce breast cancer mortality; however, many factors impact adherence to screening. Findings from the 2022 Behavioral Risk Factor Surveillance System (BRFSS) showed that, among women aged 40–74 years, the percent of those receiving a mammogram within the previous 2 years was 61.5% for American Indian or Alaska Native women, followed by Hispanic (74.3%), Non-Hispanic White (76.6%), Asian, Native Hawaiian, or other Pacific Islander (76.8%), and Non-Hispanic Black women (82.9%).^6^ Similarly, data from the 2023 National Health Interview survey (NHIS) demonstrated that age-standardized estimates of mammography use among women aged 40–74 years remained relatively stable, with 76.2% in 2019, 75.6% in 2021, and 79.8% in 2023.^7^

Based on Centers for Disease Control and Prevention data from the year 2019 showed that among women over the age of 65 years who received a mammogram, 77.5% were 400% below the national poverty level, 40.6% were uninsured, and 54.5% did not finish a high school education.^8^ Research from 2006 to 2008 of women age 65 showed that strong predictors for mammogram adherence included living with at least one chronic disease, having a higher number of visits to a primary care provider, and being married.^9^ Research based on data from the 2000 NHIS stated that the specific reasons women gave for not receiving a mammogram included lack of access, no recommendation from providers, fewer years of education, an income less than $20,000, and no family history of breast cancer.^10^ Other characteristics of non-screeners were age younger than 50 years, Hispanic ethnicity, and being born outside the United States.^10^ A longitudinal research study conducted in a mainly non-Hispanic white women showed that having self-perceived good health, being dissatisfied with past mammography experiences, and having one or more comorbidities were barriers to receive screening care.^11^

A Papanicolaou test (Pap test) is a screening tool used to observe abnormal cells in the cervix that could potentially lead to cervical cancer, a health concern that affects about 0.7% of women throughout their lifetimes.^1^ Hispanic women are disproportionally affected by cervical cancer, with an incidence of 9.7 per 100,000 women affected in the year of 2019.^1^ Findings from the BRFSS for the years 2016, 2018, and 2020 showed that the proportion of Hispanic women aged 30–64 years who were up-to-date with cervical cancer screening was 84.6% in 2016, 86.1% in 2018, and 82.8% in 2020.^12^ Current USPSTF screening guidelines are to begin Pap testing at age 21 and end at age 64, if an individual has had 3 consecutive negative results within the last 10 years; however, if this criteria is not met, screening should continue past age 65.^13^ Though screening guidelines recommend discontinuing Pap testing after the age of 65, a cross-sectional study based on Medicare expenditures from 2019 show that more than 1.3 million women older than 65 received cervical cancer screening accounting for 41% of cytology performed that year.^14^ Another study found that 24%–65% of women do not meet the criteria for cervical cancer screening cessation at 65 years old due to factors including inadequate or unknown previous screening.^14,15^ In addition, a survey from 2018 found that 25% of clinicians surveyed would continue to perform cervical cancer screening beyond the age of 65 due to concerns of missing cancer.^16^ The society for General Internal Medicine also recommends continuing cancer screening in individuals with a life expectancy greater than 10 years.^14,17^

Although Pap testing is an effective screening tool, the percent of women overdue for their screening has increased from 14.5% in 2015 to 23% in 2019.^18^ A random sample cross-sectional study of Hispanic and African American women found that the factors associated with lower Pap test screening were being older than 60 years, having Hispanic ethnicity, fewer years of education, inability to speak English, and no continuity of care with the same primary care provider.^19^ Additional barriers to receiving screenings were: patients not knowing they needed testing, no access to a physician, cost or no access to insurance, logistic barriers such as having more important obligations, living in a rural area, minority status, and sexual orientation.^20^ On the other hand, factors reported to be associated with higher Pap test screening rates included having a greater number of visits with a care provider, being insured, having a healthy body mass index (BMI), and having more years of education with knowledge of cervical cancer.^21^

Older Mexican American women are highly impacted by both breast and cervical cancer. In Hispanic women aged 65 and up, screening for breast and cervical cancer has been reported to be 60.0% and 69.0%, respectively.^22^ Between the years 2000 and 2021, the Hispanic population was the fastest growing (6.3%) minority group, reaching a total of 18.9% of the overall US population.^23^ During the same time frame, the percentage of adults over 65 years increased from 12.4% to 16.8%.^23^

The health of Mexican Americans is impacted by factors that include socioeconomic status, language barriers, healthcare access, education, and occupation, which are characterized as social determinants of health. When compared to non-Hispanic Whites living in the United States, Mexican Americans are 20 times less likely to speak proficient English, 4 times less likely to finish high school, and twice as likely to live in poverty.^24^ In addition, 70% of Hispanic adults have seen a healthcare provider in the past 12 months compared to 82% of US adults overall.^25^ Though previously discussed literature has determined factors that affect mammogram and Pap test screening adherence, few focus solely on the Mexican American population. Together, these factors can hinder access to quality healthcare, leading to poor outcomes in this patient population. Therefore, the objective of this study is to examine the predisposing, enabling, and need factors associated with mammography and Pap tests among Mexican American women aged 67 years and older over 13 years of follow-up.

## Material and methods

### Sample selection

Data were from the Hispanic Established Population for the Epidemiologic Study of the Elderly (HEPESE), a longitudinal study of Mexican American older adults aged 65 years and older residing in 5 southwestern US states (Arizona, California, Colorado, New Mexico, and Texas). The HEPESE cohort originally consisted of 3,050 Mexican Americans interviewed in 1993/1994. After providing informed consent, the participants were interviewed every 2 or 3 years by trained bilingual interviewers in the language of the participant’s choice (English or Spanish). Information on the survey questionnaire and datasets is available at the National Archive of Computerized Data on Aging.^26^ The present study used data collected from Wave 2 (1995/1996) (referred as baseline), Wave 3 (1998/1999), Wave 4 (2000/2001), Wave 5 (2004/2005) to Wave 6 (2007/2008). The prior wave interview was not included because the survey did not collect information on mammogram or Pap smear test. Inclusion criteria: Mexican American women aged 67 years and older without missing information in any of the variables included in the analysis (*N* = 912). Exclusion criteria: participants with missing information on mammogram (*n* = 10), Pap test (*n* = 58), or both tests (*n* = 50), with the remaining 409 excluded because of missing information in any of the independent variables, leaving a final sample of 912 (Supplemental Figure 1). Sample size was determined at baseline as part of the original cohort design. Additional power calculations were not conducted for follow-up waves, as the study utilized available longitudinal data and post hoc power analysis is not recommended.^27,28^

Compared to those included, those excluded were significantly more likely to be older, unmarried, have fewer years of education, and be foreign-born; to experience financial strain; to report hypertension, heart attacks, stroke, hip fractures, depressive symptoms, and limitations in activities of daily living (ADLs); and to have a lower BMI, perform lower on the Short Physical Performance Battery (SPPB), and have lower handgrip strength. Oral consent was approved by the Institutional Review Board (IRB # 92-85). Oral consent was obtained from all participants at each wave of interviews. The reporting of this study conforms to the Strengthening the Reporting of Observational Studies in Epidemiology (STROBE) statement.^29^

### Measurements

The selection of the variables included in the analysis followed the Andersen Behavioral Model of Health Services.^30,31^

### Independent variables

*Predisposing factors* included age, age at menopause (<45 years (early), 45–54 years (natural), and ⩾55 years (late)), formal years of education, marital status (married vs unmarried), living status (alone vs not alone), and nativity (US-born vs foreign-born).

*Enabling factors* included language of interview (English vs Spanish); financial strain assessed by asking participants “How much difficulty do you have in meeting monthly payments on your bills—a great deal, some, a little, or none?” with financial strain categorized as a great deal/some versus no financial strain categorized as little/none; and physician visits assessed by asking participants how many times in the past 12 months they had visited a medical doctor (0 vs ⩾ 1 visits).

*Need factors* included current smoking (Yes vs No); BMI calculated by participants’ measured weight in kg divided by their measured height in m^2^; self-reported medical conditions (hypertension, arthritis, diabetes, heart attack, stroke, hip fracture, or cancer); depressive symptoms measured with the Center for Epidemiologic Studies Depression Scale (CES-D ⩾ 16)^32,33^; cognitive function assessed utilizing the Mini Mental Status Examination (MMSE)^34^; ADL disability assessed using seven items from the Katz ADL scale (walking across a room, bathing, grooming, dressing, eating, moving from a bed to chair, and using a toilet)^35^; physical function assessed with the SPPB test (8-foot walk, standing balance, and 5 repeated chair stands), with scores ranging from 0 to 12 where higher scores indicated better physical function^36^; and muscle strength measured by handgrip strength in kg.^37^ All independent variables were obtained at each wave of interview.

### Outcome variables

Mammogram and Pap test status was determined through self-reporting. Participants were asked: “In the last 2 years, have you had a mammogram (that is, an X-ray of your breasts)?” “In the last 3 years, have you had a Pap test?” Participants were considered to have screening if they answered “yes.” Outcome variables were obtained at each wave of interview. The agreement between self-reported mammogram screening and medical records has been estimated to range from 0.73 to 0.82, with a sensitivity of 93.8%. For Pap smear tests, the agreement was reported at 81%, with a sensitivity of 94.7%.^38,39^

### Statistical analysis

Chi-square, *t*-test, and Fisher exact tests were performed to examine the baseline descriptive characteristics of the sample by mammogram and Pap screening test status. Generalized Estimating Equation (GEE) models were used to estimate the odds ratio (OR) and 95% confidence interval (CI) of having a mammogram and/or Pap test as a function of predisposing, enabling, and need factors. The models used a logit link binomial distribution. To account for repeated measures of participants we determined the most appropriate correlation structure by comparing several options, including independent, exchangeable, unstructured, and autoregressive (AR-1) using the Quasi-likelihood under the Independence model Criterion (QIC). The AR(1) structure provided the best model fit according to QIC and was therefore selected.

GEE allows for the inclusion of all available observations from participants across follow-up visits, making efficient use of partially complete data under the assumption that data are missing completely at random or missing at random. All non-missing pairs of repeated measures from the same individual contribute to the estimation of the working correlation structure. This approach helps reduce the loss of information due to incomplete follow-up while accounting for the correlation of repeated measures within individuals.^40–42^ The AR(1) working correlation structure also accommodates varying time intervals between repeated measures by assuming stronger correlation between observations closer in time. All variables were used as time varying (potential to change from interview to interview) except for age at menopause, education, and nativity, which were considered fixed. For example, if a participant did not report hypertension at baseline but reported it at the first follow-up, the participant would be categorized as having hypertension from that point onward. Two additional analyses were performed: (1) analyses were repeated without excluding participants with missing information on any of the included variables (*N* = 1439); and (2) analyses were stratified by age category (<75 vs ⩾75 years). Participants who died, refused to participate, or were lost to follow up were included until their last interview date over the 13 years of follow-up. Statistical analyses were performed by utilizing the SAS System for Windows version 9.4 (SAS Institute, Inc., Cary, NC, United States).

## Results

Table 1 presents the baseline descriptive characteristics of the sample overall and by self-reported mammogram and Pap test status. The overall average age of the sample was 74.6 ± 5.8 years, with menopause occurring early in 34.5%, natural in 54.7%, and late in 10.8%. About 41% of the participants were married, 30% lived alone, 41.1% were US-born, 80.2% were interviewed in Spanish, 62.7% reported financial strain, and the mean years of education was 5.1 ± 3.9 years. The most frequently reported medical conditions were arthritis (55.0%), hypertension (53.4%), and diabetes (28.7%). About 15% reported high depressive symptoms and 7% reported ADL disability. About 81% had at least one medical visit in the past 12 months, 61.7% received Pap testing, and 66.5% mammogram testing. Mean MMSE, SPPB, and handgrip scores were 24.7 ± 3.9, 8.2 ± 2.8, and 19.6 ± 5.3 respectively.

**Table 1.** Baseline descriptive characteristics of the sample overall and by mammogram and Papanicolaou (Pap) test among older Mexican American women (*N* = 912).

Variables	Overall	Mammogram test		*p*-Value	Pap test		*p*-Value
		Yes, *N* (%)	No, *N* (%)		Yes, *N* (%)	No, *N* (%)	
Total	912	606 (66.5)	306 (33.5)		563 (61.7)	349 (38.3)	
Predisposing factors							
Age (years), mean ± SD	74.6 ± 5.8	73.9 ± 5.4	75.7 ± 6.2	<0.0001	73.79 ± 5.3	75.6 ± 6.3	<0.0001
Age at menopause				0.2224			0.0013
Early (<45 years)	315 (34.5)	198 (32.7)	117 (38.2)		170 (30.2)	145 (41.6)	
Natural (45–54 years)	499 (54.7)	339 (55.9)	160 (52.3)		324 (57.6)	175 (50.1)	
Late (⩾55 years)	98 (10.8)	69 (11.4)	29 (11.4)		69 (12.3)	69 (8.3)	
Marital status (married)	374 (41.0)	263 (43.4)	111 (36.3)	0.0389	250 (44.4)	124 (35.5)	0.0081
Nativity (foreign-born)	375 (41.1)	242 (39.9)	133 (43.5)	0.3063	213 (37.8)	162 (46.4)	0.0104
Education (years), mean ± SD	5.1 ± 3.9	5.4 ± 4.0	4.3 ± 3.6	<0.0001	5.8 ± 3.9	3.85 ± 3.4	<0.0001
Enabling factors							
Language of interview (Spanish)	731 (80.2)	472 (77.9)	259 (84.6)	0.0158	418 (74.3)	313 (89.7)	<0.0001
Financial strain	572 (62.7)	362 (59.7)	210 (68.6)	0.0087	323 (57.4)	249 (71.4)	<0.0001
Lives alone	274 (30.0)	183 (30.2)	91 (29.7)	0.8864	173 (30.7)	101 (28.9)	0.5669
MD visits	814 (89.3)	568 (93.7)	246 (80.4)	<0.0001	526 (93.4)	288 (82.5)	<0.0001
Need factors							
Medical conditions							
Hypertension	487 (53.4)	349 (57.6)	138 (45.1)	0.0004	330 (58.6)	157 (45.0)	<0.0001
Arthritis	502 (55.0)	367 (60.6)	135 (44.1)	<0.0001	350 (62.2)	152 (43.6)	<0.0001
Diabetes	262 (28.7)	178 (29.4)	84 (27.5)	0.5447	175 (31.1)	87 (24.9)	0.0459
Heart attack	56 (6.1)	45 (7.4)	11 (3.6)	0.0229	37 (6.6)	19 (5.4)	0.4905
Stroke	57 (6.3)	44 (7.3)	13 (4.3)	0.0760	40 (7.1)	17 (4.9)	0.1756
Hip fracture	12 (1.3)	10 (1.7)	2 (0.7)	0.2124	6 (1.1)	6 (1.7)	0.3999
Cancer	62 (6.8)	51 (8.4)	11 (3.6)	0.0063	43 (7.6)	19 (5.4)	0.2009
BMI (kg/m^2^), mean ± SD	29.1 ± 5.7	29.5 ± 5.8	28.3 ± 5.5	0.0026	29.6 ± 5.8	28.4 ± 5.5	0.0015
Current smoker	69 (7.6)	48 (7.9)	21 (6.9)	0.5683	43 (7.6)	26 (7.5)	0.9170
Depressive symptoms (CES-D ⩾ 16)	134 (14.7)	104 (17.2)	30 (9.8)	0.0030	98 (17.4)	36 (10.3)	0.0033
MMSE	24.7 ± 3.9	25.1 ± 3.9	23.9 ± 3.9	<0.0001	25.4 ± 3.7	23.55 ± 3.9	<0.0001
Pain	407 (44.6)	290 (47.9)	117 (38.2)	0.0058	268 (47.6)	139 (39.8)	0.0217
ADL disability	64 (7.0)	39 (6.4)	25 (8.2)	0.3330	38 (6.8)	26 (7.5)	0.6874
SPPB, mean ± SD	8.2 ± 2.8	8.15 ± 2.8	8.0 ± 2.9	0.4456	8.2 ± 2.9	7.9 ± 2.8	0.1771
Handgrip strength (kg), mean ± SD	19.6 ± 5.3	19.8 ± 5.4	19.1 ± 5.1	0.0594	19.9 ± 5.2	19.1 ± 5.3	0.0313

SD: standard deviation; MD: medical doctor; BMI: body mass index; CES-D: Center for Epidemiologic Studies Depression Scale; MMSE: mini mental state examination; ADL: activities of daily living; SPPB: short physical performance battery.

Women who did not receive a mammogram test were significantly more likely to be older, unmarried, and interviewed in Spanish; to have fewer years of education; to report financial strain and pain; and to report less hypertension, arthritis, heart attack, cancer, high depressive symptoms, ADL disability, physician visits, Pap test screening, lower BMIs, and higher MMSE scores compared to women who received a mammogram test (Table 1). Women who did not receive a Pap test were significantly more likely to be older, to have had early menopause, be unmarried, be foreign-born, and be interviewed in Spanish; to have fewer years of education; to report financial strain and pain; to be less likely to report hypertension, arthritis, diabetes, depressive symptoms, physician visits, and mammogram screening; and to have lower MMSE scores, lower BMIs, and lower handgrip strength compared to women who received a Pap test (Table 1).

**Figure 1.**
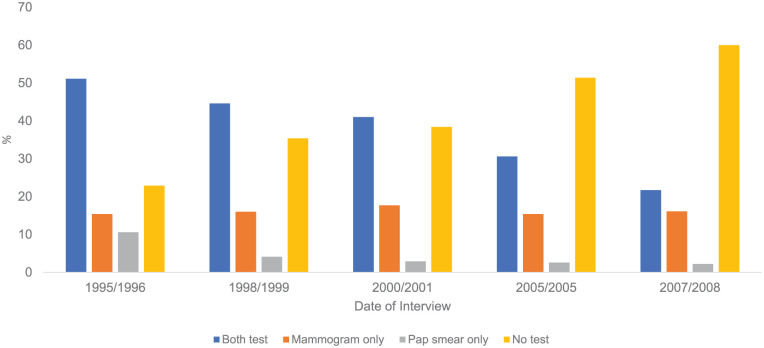
Percent of mammogram and Papanicolaou (Pap) test screening over time among Mexican American women.

Figure 1 presents the percent of participants receiving screenings from baseline to Wave 6. The percent of participants receiving both mammogram and Pap tests decreased from 51.1% to 21.7%. The percent of those receiving a Pap test only decreased from 10.6% to 2.2%, while the percent of those receiving a mammogram test only decreased from 17.7% to 15.4%. The percent of those not receiving both tests increased from 22.9% at baseline to 60% at Wave 6. Supplemental Figure 2 presents the percent of participants receiving screenings from baseline to Wave 6 stratified by age. In both age categories (<75 and ⩾75 years), the percent of screening tests decreased over time and more pronounced in those ⩾75 years.

Table 2 presents the OR and 95% CI of receiving a mammogram, Pap test, and both tests over time as a function of predisposing, enabling, and need factors (*N* = 912). The odds of receiving a mammogram per year was 0.95 (95% CI = 0.93–0.98), receiving a Pap test was 0.91 (95% CI = 0.88–0.93), and receiving both tests was 0.94 (95% CI = 0.91–0.96). For mammogram test, higher level of education (OR = 1.04, 95% CI = 1.01–1.07), having at least one physician visit (OR = 2.17, 95% CI = 1.61–2.91), hypertension (OR = 1.45, 95% CI = 1.20–1.76), arthritis (OR = 1.27, 95% CI = 1.04–1.54), stroke (OR = 1.53, 95% CI = 1.02–2.28), and cancer (OR = 1.54, 95% CI = 1.03–2.31) were factors associated with greater odds of receiving a mammogram test over time. Older age (OR = 0.96, 95% CI = 0.94–0.98) and financial strain (OR = 0.83, 95% CI = 0.70–0.99) were factors associated with lower odds of receiving a mammogram test over time.

**Table 2.** Generalized estimating equation models for mammogram and Papanicolaou (Pap) tests among older Mexican American women (*N* = 912).

Variables	Mammogram test, OR 95% (CI)	*p*-Value	Pap test, OR 95% (CI)	*p*-Value	Both tests, OR 95% (CI)	*p*-Value
Time (years)	0.95 (0.93–0.98)	<0.0001	0.91 (0.88–0.93)	<0.0001	0.94 (0.91–0.96)	<0.0001
Predisposing factors						
Age (years)	0.96 (0.94–0.98)	<0.0001	0.96 (0.94–0.98)	0.0001	0.96 (0.94–0.98)	0.0004
Age at menopause						
Early (<45)	0.86 (0.69–1.06)	0.1711	0.69 (0.56–0.86)	0.0008	0.71 (0.58–0.89)	0.0021
Natural (45–54)	Reference		Reference			
Late (⩾55)	1.10 (0.77–1.57)	0.5976	1.28 (0.86–1.74)	0.2516	1.15 (0.82–1.61)	0.4269
Marital status (married)	1.15 (0.92–1.46)	0.2182	1.11 (0.88–1.41)	0.3817	1.17 (0.93–1.47)	0.1865
Nativity (foreign-born)	1.01 (0.82–1.25)	0.9340	1.02 (0.83–1.26)	0.8260	0.95 (0.78–1.18)	0.6883
Education (years)	1.04 (1.01–1.07)	0.0044	1.05 (1.02–1.08)	0.0016	1.04 (1.01–1.07)	0.0030
Enabling factors						
Language of interview (Spanish)	0.99 (0.77–1.27)	0.9489	0.71 (0.56–0.91)	0.0061	0.79 (0.62–1.01)	0.0550
Financial strain	0.83 (0.70–0.99)	0.0354	0.84 (0.70–1.00)	0.0554	0.85 (0.71–1.01)	0.0643
Lives alone	1.23 (0.98–1.55)	0.0762	1.20 (0.96–1.51)	0.1132	1.13 (0.90–1.41)	0.2971
MD visits	2.17 (1.61–2.91)	<0.001	2.09 (1.52–2.87)	<0.001	1.85 (1.33–2.56)	0.0002
Need factors						
Medical conditions						
Hypertension	1.45 (1.20–1.76)	0.0001	1.18 (0.97–1.43)	0.0909	1.26 (1.04–1.51)	0.0173
Arthritis	1.27 (1.04–1.54)	0.0177	1.25 (1.02–1.53)	0.0282	1.31 (1.07–1.60)	0.0093
Diabetes	1.05 (0.86–1.30)	0.6156	1.14 (0.93–1.39)	0.2146	1.09 (0.89–1.33)	0.3837
Heart attack	1.25 (0.84–1.84)	0.2721	1.26 (0.87–1.81)	0.2298	1.25 (0.87–1.78)	0.2368
Stroke	1.53 (1.02–2.28)	0.0394	1.27 (0.88–1.83)	0.1984	1.28 (0.90–1.84)	0.1744
Hip fracture	1.26 (0.72–2.19)	0.4160	0.86 (0.50–1.49)	0.5955	0.97 (0.58–1.64)	0.9116
Cancer	1.54 (1.03–2.31)	0.0367	1.05 (0.72–1.53)	0.7886	1.10 (0.75–1.61)	0.6260
BMI (kg/m^2^)	1.02 (0.99–1.04)	0.0943	1.01 (0.99–1.03)	0.3098	1.01 (0.99–1.03)	0.1968
Current smoker	0.87 (0.60–1.26)	0.4534	0.81 (0.57–1.14)	0.2273	0.71 (0.50–1.01)	0.0605
Depressive symptoms (CES-D ⩾ 16)	1.04 (0.83–1.31)	0.7274	1.19 (0.94–1.50)	0.1550	1.18 (0.94–1.49)	0.1501
MMSE	1.02 (0.99–1.04)	0.1094	1.03 (1.01–1.05)	0.0050	1.02 (0.99–1.04)	0.0775
Pain	0.99 (0.84–1.18)	0.9434	0.98 (0.81–1.17)	0.7841	1.01 (0.85–1.21)	0.8809
ADL disability	0.82 (0.63–1.06)	0.1212	0.97 (0.73–1.28)	0.8072	0.85 (0.64–1.13)	0.2705
SPPB	0.99 (0.96–1.03)	0.9279	1.01 (0.98–1.05)	0.4512	1.01 (0.98–1.04)	0.5127
Handgrip strength (kg)	1.02 (0.99–1.04)	0.0639	1.01 (0.99–1.03)	0.1481	1.02 (1.01–1.04)	0.0420

OR: odds ratio; CI: confidence interval; MD: medical doctor; BMI: body mass index; CES-D: Center for Epidemiologic Studies Depression Scale; MMSE: mini mental state examination; SPPB: short physical performance battery; ADL: activities of daily living.

Higher level of education (OR = 1.05, 95% CI = 1.02–1.08), having at least one physician visit (OR = 2.09, 95% CI = 1.52–2.87), arthritis (OR = 1.25, 95% CI = 1.02–1.53), and higher MMSE score (OR = 1.03, 95% CI = 1.01–1.05) were factors associated with greater odds of receiving a Pap test over time. Older age (OR = 0.96, 95% CI = 0.94–0.98), early menopause (OR = 0.69, 95% CI = 0.56–0.86), and being interviewed in Spanish (OR = 0.71, 95% CI = 0.56–0.91) were factors associated with lower odds of receiving a Pap test over time (Table 2).

Higher level of education (OR = 1.04, 95% CI = 1.01–1.07), having at least one physician visit (OR = 1.85, 95% CI = 1.33–2.56), hypertension (OR = 1.26, 95% CI = 1.04–1.51), arthritis (OR = 1.31, 95% CI = 1.07–1.60), and high performance on handgrip strength (OR = 1.02, 95% CI = 1.01–1.04) were factors associated with greater odds of receiving both tests. Older age (OR = 0.96, 95% CI = 0.94–0.98) and early menopause (OR = 0.71, 95% CI = 0.58–0.89) were factors associated with lower odds of receiving both tests over time (Table 2).

Supplemental Table 1 shows the OR and 95% CI of receiving a mammogram, Pap test, and both tests over time as a function of predisposing, enabling, and need factors among women <75 years. High level of education, having at least one physician visit, and hypertension were factors associated with grater odds of receiving a mammogram test over time. Any physician visit was the only factor associated with greater odds of receiving a Pap test over time. High level of education, any physician visit, hypertension, and depressive symptoms were factors associated with greater odds of receiving both tests over time.

Supplemental Table 2 shows the OR and 95% CI of receiving a mammogram, Pap test, and both tests over time as a function of predisposing, enabling, and need factors among women ⩾75 years. Any physician visit, stroke, cancer, and higher BMIs were factors associated with greater odds of receiving a mammogram test. High level of education, any physician visit, arthritis, and higher score on the MMSE were factors associated with greater odds of receiving a Pat test. High level of education, any physician visit, arthritis, and high performance in handgrip strength were associated with greater odds of receiving both tests, whereas early menopause was associated with 37% lower odds of receiving both tests over time.

## Discussion

This study examined factors associated with receipt of mammogram and/or Pap screening tests over time in older Mexican American women. Our analysis showed a decline in Pap test screening over time, while mammogram rates remained relatively steady. Factors associated with receiving both screening tests included higher levels of education, having any physician visits, hypertension, arthritis, and high performance in handgrip strength. In contrast, older age and early menopause were associated with lower odds of receiving both screening tests over time.

Our findings regarding the decline in Pap test screenings over time are consistent with the USPSTF recommendations from 1996 (the beginning of this study) to screen every 3 years until the age of 65.^43^ Additionally, in 1996, the USPSTF recommended mammograms every 1–2 years from age 50 to 69, later extended the upper age limit to 75 years in 2009.^3,43^ Many women in our survey fell well within this recommended age range, which may explain the relatively stable rates of mammogram use over time. Currently, the USPSTF recommends biennial mammography for women aged 50–74 years, and states that evidence is insufficient to assess the balance of benefits and harms for women older than 75.^2^ For cervical cancer, screening is recommended to stop at age 65, provided prior screening was adequate and risk is low.^13^ These evolving guidelines help contextualize the observed trends in screening rates across age groups.

Several explanations may clarify why certain factors are associated with mammogram and Pap test screening. The association between higher level of education and adherence to health screenings may be associated with health literacy, which measures an individual’s ability to understand basic healthcare information (written and verbal) and the services needed to make appropriate decisions surrounding one’s health.^44^ The association of interviewing in Spanish with lower screening rates is unsurprising, since research has shown that language barriers and cultural differences negatively impact cancer screening care among immigrant patients.^45^ Regarding financial strain, all participants in this study were covered by Medicare; however, financial strain may prevent access by making people unable to pay to travel to appointments, pay insurance co-pays, or afford time off work for screening appointments. Visiting a healthcare provider and having medical conditions such as arthritis, hypertension, stroke, and cancer, which may serve as proxies, are strongly associated with receiving cancer screening.^46,47^ The most likely explanation is that regular interaction with healthcare providers for chronic disease management allows the healthcare provider to recommend and follow up on preventive screenings. While women with arthritis show higher odds of receiving cancer screening, this practice does not translate to lower cancer mortality. In fact, studies have shown that the cancer-related mortality in women with rheumatoid arthritis is comparable to that of the general population.^48–50^

Our findings regarding comorbid conditions such as hypertension, arthritis, stroke, and cancer align with some previous studies, although not all.^9,46,51,52^ For example, a study using the 2018 and 2019 BRFSS found that women aged 50–74 with hypertension, arthritis, and cancer had higher adjusted risk ratios of completing a breast cancer screening.^47^ In contrast, another study found that women aged 65 years and older with at least one chronic disease had 3% lower odds of receiving a mammogram test.^9^ Similarly, Heflin et al. reported that, among women aged 65 years and older, hypertension was associated with 41% greater odds of receiving a Pap smear test but not a mammogram.^51^

Gaps exist in the literature about factors associated with mammogram and Pap test screening in older Mexican American women; however, some of our findings are consistent with previous studies conducted in other racial and ethnic groups. As mentioned, studies have found that fewer years of education, being Hispanic, being born outside the United States, being older than 60 years, not speaking English, and not having continuous medical care are negative predictors of obtaining screening tests.^10,19^ Education was another positive predictor for screening. A meta-analysis by Damiani et al.^53^ concluded that women with the highest levels of education were 1.6 and 1.96 times, respectively, to be screened for mammogram and Pap test. Although our results are consistent with these studies, others included younger women and some did not stratify by race and ethnicity, making direct comparisons difficult.

### Study limitations

There are some limitations to this study. First, mammogram and Pap test screening were self-reported by participants and not obtained from medical charts. In addition, the participants were asked if they had received a mammogram or Pap test over the past 2 or 3 years, respectively. The specific time frames may have impacted the reliability of the data due to recall bias. Second, our findings are generalizable only to the Mexican American population living in the Southwestern Unites States and may not reflect the experience of other Hispanic groups. For example, women from Cuba are less likely to have a mammogram screening than Mexican American women, while those from Puerto Rico are more likely.^54^ Third, information about history of mammogram or Pap test screening in middle age was not available in the survey. Fourth, while a power analysis was conducted at baseline, we did not perform additional power calculations for follow-up waves. This is a limitation, although the study was based on available data from a well-established cohort, and post hoc power analysis is generally discouraged. Despite these limitations, the strengths of our study include the use of a large population of Mexican American women with information from 13 years of follow-up, which allows for analysis of changes over time in sociodemographics and health characteristics.

## Conclusions

Our study found that Spanish-speaking Mexican American women had 29% lower odds of receiving a Pap smear test compared to their English-speaking counterparts, after controlling for all variables. Financial strain was also associated with a 17% reduction in the odds of receiving a mammogram. In contrast, higher educational attainment, presence of hypertension or arthritis, having any physician visit, and high performance in handgrip strength were all associated with greater odds of receiving both screenings. Women who experienced early menopause had 29% lower odds of receiving both tests.

These findings highlight the need for targeted interventions to improve screening rates in this population. Potential strategies include establishing bilingual or Spanish-speaking women’s health clinics to reduce language and cultural barriers, as well as offering weekend or mobile screening services to accommodate those with limited transportation or inflexibility work schedules. Overall, our results emphasize the importance of addressing systemic barriers to enhance access to preventive care and reduce cancer disparities in older Mexican American women.

## Supplemental Material

sj-docx-1-whe-10.1177_17455057261421727 – Supplemental material for Factors associated with mammogram and Papanicolaou testing among Mexican American older women Supplemental material, sj-docx-1-whe-10.1177_17455057261421727 for Factors associated with mammogram and Papanicolaou testing among Mexican American older women by Emma Rowlinson and Soham Al Snih in Women's Health
