# Supplementary material for: Factors associated with mammogram and Papanicolaou testing among Mexican American older women
Source: Womens Health (Lond). 2026 Feb 19;22:17455057261421727. doi: 10.1177/17455057261421727 (PMC12924983; doi:10.1177/17455057261421727)
Supplement: sj-docx-1-whe-10.1177_17455057261421727 – Supplemental material for Factors associated with mammogram and Papanicolaou testing among Mexican American older women [file sj-docx-1-whe-10.1177_17455057261421727.docx]

**Supplemental Figure 2. Percent of mammogram and Papanicolaou (Pap) test screening over time among Mexican American women aged < 75 (A) and ≥ 75 years (B).**

**Supplementary - Table 1. Generalized estimating equation models for mammogram and Papanicolaou (Pap) tests among older Mexican American women aged < 75 years (N=532).**

| **Variables** | **Mammogram test**  **OR 95% (CI)** | **p-value** | **Pap test**  **OR 95% (CI)** | **p-value** | **Both tests**  **OR 95% (CI)** | **p- value** |
| --- | --- | --- | --- | --- | --- | --- |
| **Time (years)** | 0.92 (0.89-0.94) | <0.0001 | 0.88 (0.85-0.91) | < 0.0001 | 0.91 (0.89-0.94) | <0.0001 |
| ***Predisposing factors*** |  |  |  |  |  |  |
| Early (< 45) | 0.94 (0.71-1.24) | 0.6643 | 0.70 (0.54-0.91) | 0.0079 | 0.75 (0.58-0.98) | 0.0376 |
| Natural (45-54) | Reference |  | Reference |  | Reference |  |
| Late (≥ 55) | 0.87 (0.52-1.45) | 0.5961 | 0.96 (0.57-1.64) | 0.8927 | 0.89 (0.55-1.46) | 0.6569 |
| Married | 1.19 (0.89-1.60) | 0.2359 | 1.15 (0.86-1.54) | 0.3436 | 1.17 (0.93-1.47) | 0.1865 |
| Foreign-born | 1.02 (0.77-1.35) | 0.8744 | 1.10 (0.84-1.44) | 0.4921 | 0.99 (0.75-1.30) | 0.9400 |
| Education (years) | 1.04 (1.01-1.11) | 0.0200 | 1.03 (0.99-1.07) | 0.0552 | 1.04 (1.00-1.07) | 0.0257 |
| ***Enabling Factors*** |  |  |  |  |  |  |
| Spanish interview | 1.00 (0.73-1.39) | 0.9781 | 0.67 (0.49-0.92) | 0.0128 | 0.78 (0.57-1.07) | 0.1248 |
| Financial Strain | 0.82 (0.65-1.03) | 0.0820 | 0.85 (0.68-1.06) | 0.1491 | 0.84 (0.67-1.05) | 0.1309 |
| Lives alone | 1.27 (0.93-1.72) | 0.1321 | 1.24 (0.92-1.68) | 0.1601 | 1.21 (0.90-1.62) | 0.2170 |
| MD Visits | 2.17 (1.52-3.09) | < 0.0001 | 1.88 (1.32-2.71) | 0.0006 | 1.83 (1.28-2.67) | 0.0016 |
| ***Need factors*** |  |  |  |  |  |  |
| **Medical conditions** |  |  |  |  |  |  |
| Hypertension | 1.52 (1.19-1.93) | 0.0007 | 1.17 (0.92-1.48) | 0.1909 | 1.29 (1.02-1.64) | 0.0330 |
| Arthritis | 1.24 (0.98-1.57) | 0.0689 | 1.17 (0.92-1.49) | 0.2081 | 1.19 (0.93-1.51) | 0.1653 |
| Diabetes | 1.04 (0.80-1.35) | 0.7665 | 1.27 (0.99-1.64) | 0.0606 | 1.17 (0.91-1.50) | 0.2267 |
| Heart Attack | 1.32 (0.74-2.33) | 0.3441 | 1.09 (0.66-1.82) | 0.7296 | 1.23 (0.75-2.03) | 0.4128 |
| Stroke | 1.25 (0.72-2.16) | 0.4268 | 1.03 (0.62-1.72) | 0.9153 | 1.09 (0.67-1.78) | 0.7398 |
| Hip fracture | 1.35 (0.64-2.84) | 0.4324 | 1.42 (0.64-3.12) | 0.3878 | 1.64 (0.77-3.51) | 0.1987 |
| Cancer | 1.42 (0.83-2.43) | 0.2066 | 1.01 (0.61-1.67) | 0.9603 | 1.00 (0.59-1.69) | 0.9910 |
| BMI (Kg/m^2^) | 1.01 (0.99-1.04) | 0.2747 | 1.01 (0.99-1.04) | 0.2773 | 1.02 (0.99-1.04) | 0.1525 |
| Current smoker | 1.01 (0.66-1.56) | 0.9578 | 0.86 (0.60-1.25) | 0.4325 | 0.83 (0.56-1.21) | 0.3217 |
| CES-D ≥ 16 | 1.13 (0.84-1.51) | 0.4378 | 1.26 (0.95-1.67) | 0.1109 | 1.34 (1.02-1.76) | 0.0329 |
| MMSE | 1.02 (0.99-1.05) | 0.0909 | 1.02 (0.99-1.05) | 0.1023 | 1.02 (0.99-1.04) | 0.2151 |
| Pain | 1.05 (0.85-1.30) | 0.6272 | 1.04 (0.83-1.30) | 0.7338 | 1.05 (0.85-1.31) | 0.6362 |
| ADL Disability | 0.82 (0.59-1.13) | 0.2192 | 0.87 (0.61-1.25) | 0.4481 | 0.78 (0.55-1.12) | 0.1823 |
| SPPB | 1.00 (0.95-1.04) | 0.8780 | 1.03 (0.99-1.07) | 0.1943 | 1.02 (0.98-1.07) | 0.2409 |
| Handgrip strength (kg) | 1.02 (0.99-1.04) | 0.0991 | 1.01 (0.99-1.04) | 0.2687 | 1.02 (0.99-1.04) | 0.1405 |

Note: OR=Odds Ratio; CI=Confidence Interval; MD=Medical Doctor; BMI=Body Mass Index; CES-D=Center for Epidemiologic Studies Depression Scale; MMSE=Mini Mental State Examination; SPPB=Short Physical Performance Battery; ADL=Activities of Daily Living.

**Supplementary - Table 2. Generalized estimating equation models for mammogram and Papanicolaou (Pap) tests among older Mexican American women aged ≥ 75 years (N=380).**

| **Variables** | **Mammogram test**  **OR 95% (CI)** | **p-value** | **Pap test**  **OR 95% (CI)** | **p-value** | **Both tests**  **OR 95% (CI)** | **p- value** |
| --- | --- | --- | --- | --- | --- | --- |
| **Time (years)** | 0.92 (0.89-0.96) | <0.0001 | 0.88 (0.84-0.93) | < 0.0001 | 0.91 (0.87-0.95) | <0.0001 |
| ***Predisposing factors*** |  |  |  |  |  |  |
| Age at menopause |  |  |  |  |  |  |
| Early (< 45) | 0.73 (0.50-1.07) | 0.1037 | 0.70 (0.48-1.02) | 0.0631 | 0.63 (0.43-0.93) | 0.0211 |
| Natural (45-54) | Reference |  | Reference |  | Reference |  |
| Late (≥ 55) | 1.31 (0.81-2.11) | 0.2656 | 1.48 (0.93-2.35) | 0.1015 | 1.40 (0.88-2.21) | 0.1549 |
| Married | 1.23 (0.84-1.79) | 0.2865 | 1.12 (0.74-1.70) | 0.5928 | 1.15 (0.77-1.71) | 0.4890 |
| Foreign-born | 0.96 (0.69-1.35) | 0.8225 | 0.90 (0.64-1.25) | 0.5205 | 0.89 (0.64-1.25) | 0.5096 |
| Education (years) | 1.04 (0.99-1.10) | 0.1020 | 1.08 (1.02-1.13) | 0.0038 | 1.06 (1.00-1.11) | 0.0365 |
| ***Enabling Factors*** |  |  |  |  |  |  |
| Spanish interview | 0.98 (0.67-1.44) | 0.9320 | 0.73 (0.50-1.06) | 0.1025 | 0.77 (0.54-1.11) | 0.1616 |
| Financial Strain | 0.88 (0.67-1.16) | 0.3589 | 0.84 (0.62-1.14) | 0.2692 | 0.88 (0.65-1.18) | 0.3853 |
| Lives alone | 1.15 (0.81-1.62) | 0.4400 | 1.14 (0.80-1.61) | 0.4744 | 1.01 (0.71-1.43) | 0.9451 |
| MD Visits | 2.20 (1.31-3.71) | 0.0031 | 2.65 (1.45-4.85) | 0.0015 | 1.87 (1.03-3.40) | 0.0386 |
| ***Need factors*** |  |  |  |  |  |  |
| **Medical conditions** |  |  |  |  |  |  |
| Hypertension | 1.34 (0.98-1.84) | 0.0697 | 1.14 (0.83-1.58) | 0.4193 | 1.17 (0.85-1.60) | 0.3309 |
| Arthritis | 1.38 (0.97-1.96) | 0.0705 | 1.51 (1.06-2.14) | 0.0212 | 1.66 (1.15-2.40) | 0.0073 |
| Diabetes | 1.09 (0.76-1.55) | 0.6409 | 0.99 (0.70-1.41) | 0.9669 | 0.97 (0.69-1.36) | 0.8533 |
| Heart Attack | 1.24 (0.72-2.12) | 0.4439 | 1.59 (0.92-2.74) | 0.0948 | 1.36 (0.81-2.30) | 0.2487 |
| Stroke | 1.91 (1.05-3.48) | 0.0335 | 1.46 (0.87-2.48) | 0.1553 | 1.45 (0.86-2.46) | 0.1634 |
| Hip fracture | 1.13 (0.52-2.43) | 0.7607 | 0.50 (0.23-1.11) | 0.0891 | 0.56 (0.27-1.15) | 0.1144 |
| Cancer | 1.85 (1.01-3.36) | 0.0448 | 1.20 (0.69-2.09) | 0.5239 | 1.32 (0.77-2.26) | 0.3159 |
| BMI (Kg/m^2^) | 1.03 (1.00-1.07) | 0.0332 | 1.01 (0.98-1.05) | 0.3907 | 1.01 (0.98-1.04) | 0.4846 |
| Current smoker | 0.66 (0.35-1.26) | 0.2085 | 0.81 (0.34-1.91) | 0.6314 | 0.50 (0.21-1.19) | 0.1162 |
| CES-D ≥ 16 | 0.96 (0.66-1.41) | 0.8520 | 1.08 (0.72-1.63) | 0.7107 | 0.97 (0.64-1.47) | 0.8850 |
| MMSE | 1.01 (0.98-1.04) | 0.4062 | 1.04 (1.01-1.08) | 0.0132 | 1.02 (0.99-1.06) | 0.1471 |
| Pain | 0.88 (0.65-1.19) | 0.3990 | 0.84 (0.61-1.14) | 0.2652 | 0.89 (0.65-1.21) | 0.4559 |
| ADL Disability | 0.82 (0.54-1.26) | 0.3688 | 1.12 (0.72-1.75) | 0.6163 | 0.97 (0.61-1.52) | 0.8823 |
| SPPB | 1.00 (0.96-1.06) | 0.7313 | 1.01 (0.98-1.05) | 0.4512 | 1.00 (0.95-1.06) | 0.9559 |
| Handgrip strength (kg) | 1.02 (0.99-1.05) | 0.1525 | 1.02 (0.99-1.06) | 0.1339 | 1.03 (1.00-1.06) | 0.0408 |

Note: OR=Odds Ratio; CI=Confidence Interval; MD=Medical Doctor; BMI=Body Mass Index; CES-D=Center for Epidemiologic Studies Depression Scale; MMSE=Mini Mental State Examination; SPPB=Short Physical Performance Battery; ADL=Activities of Daily Living.

**Supplemental Figure 1. Flow chart of participant selection.**

**Wave 5 (2004/05)**

Interviewed, n=514

Deaths, n=145

Refused/lost to follow-up, n=119

**Baseline Sample**

(1995/96)

N=1439

**Missing information:**

Mammogram test (n=10)

Papanicolaou test (58)

Both tests (n=50)

Any independent variables at baseline (n=409)

**Wave 3 (1998/99)**

Interviewed, n=779

Deaths, n=73

Refused/lost to follow-up, n=60

**Wave 4 (2000/01)**

Interviewed, n=700

Deaths, n=61

Refused/lost to follow-up, n=78

**Final Sample**

(1995/96)

N=912

**Wave 6 (2007/08)**

Interviewed, n=427

Deaths, n=100

Refused/lost to follow-up, n=106

**3-Year Follow Up**

**5-Year Follow Up**

**9-Year Follow Up**

**Eligibility**

**Included**

**13-Year Follow Up**
